# Asynchronous Online Instruction Leads to Learning Gaps When Compared to a Flipped Classroom

**DOI:** 10.1007/s10956-022-09988-7

**Published:** 2022-08-11

**Authors:** Jamie Jensen, Clara M. Smith, Robert Bowers, Mahealani Kaloi, T. Heath Ogden, Kirkham A. Parry, Joshua S. Payne, Porter Fife, Emily Holt

**Affiliations:** 1 Department of Biology, Brigham Young University, Provo, UT USA; 2 Department of Biology, Utah Valley University, Orem, UT USA; 3 Continuing Education Research, Brigham Young University, Planning, & Assessment, Provo, UT USA; 4 School of Biological Sciences, University of Northern Colorado, Greely, CO USA

**Keywords:** Online education, Flipped classroom, Learning environment, Biology education

## Abstract

With the gradual shift to online education models that has taken place in recent decades, research has sought to understand the nuances of student performance in an online model in comparison to more traditional in-person modalities. However, the effects of instructional modality have been difficult to determine given the many variables that exist in course design between these methods. In this study, we attempt to determine the efficacy of asynchronous online instruction by comparing two nearly equivalent courses. The first course was a flipped classroom, a recent and well-studied hybrid model of instruction. The second was an asynchronous fully online course that contained all the same instructional elements as the in-person course but lacked any student or instructor interaction. Student performance was tracked at both a highly-selective private institution and an open-enrollment public institution. Results show that students’ performance drops in an asynchronous online course compared to an equivalent in-person experience. Several potential hypotheses are put forth to explain a change in performance that can potentially shape the design of online instruction.

## Introduction

Higher education has evolved over recent decades to effectively use technology and move many classes to a full or partial online format. Between 2012 and 2015, online enrollment in the USA has increased 30% (Allen & Seaman, 2015), and as of 2015, 43.1% of undergraduate students have taken at least one online course (National Center for Education Statistics, 2018). With the COVID-19 pandemic, higher education experienced the abrupt pivot to remote teaching. Now, more than ever, it is imperative that we continue to investigate the effects of online instruction on student learning and compare learning gains to in-person instruction.

Online instruction can take many forms. Prior to the advent of the internet, distance education (DE) took the form of correspondence education, where materials were exchanged between student and instructor via mail (Kentnor, 2015). As defined by Gallagher and McCormick (1999), DE takes place when there is at least a semi-permanent separation between the learner and the instructor. In the 1990s, Internet capabilities allowed for many different forms of DE (Phipps & Merisotis, 2000) and further contributed flexibility to in-person courses (e.g., blended, hybrid, flipped). A blended course uses digital resources to supplement learning that are not meant to replace in-person instruction; whereas a hybrid course implements digital content to replace part of the in-person instructional time (Siegelman, 2020). A flipped classroom can be blended or hybrid, but it may also lack digital materials (see Jensen et al., 2018). Regardless, a flipped course uses curriculum designed so that students learn basic materials asynchronously before class (most traditional through digital media but can also be through textbook learning) and then practice applying that content in an in-person setting (Hamdan et al., 2013). A fully online course, on the other hand, is one in which there is a permanent separation of the learner and instructor and all course interactions, whether synchronous or asynchronous, take place in a digital format (Means et al., 2010). The sheer multitude of variations of an online course design undoubtedly contributes to the mixed findings the education research community reports in comparing online with in-person instruction.

Beyond differences in online versus in-person course delivery, a host of other influential factors such as learner time-on-task, pedagogical approach, or curricular materials (Means et al., 2010) confound findings across studies. In fact, Zhao et al. (2005) found effect sizes comparing online to in-person instruction were dependent upon instructor identity, instructor involvement, the outcome measure, student education level, content area, level of instruction, degree of synchronous interactions, and degree of in-person teaching. The purpose and contribution of this study is to control for many of these features by comparing a fully online asynchronous course (i.e., where no synchronous interaction takes place) to a flipped version of an in-person course while maintaining equivalent pedagogical approaches, instructors, time-on-task, and curricular materials. Our guiding research question was, are student academic outcomes equivalent when simply transferring the in-person assignments, activities, and assessments of a flipped classroom directly to a fully online asynchronous format?

### Comparisons of Online and In-Person Instruction

Many meta-analyses have been performed on DE methods. However, to determine the effectiveness of online education in comparison to in-person instruction, we only summarize those meta-analyses that included studies that directly compared online classrooms with in-person classrooms and excluded studies comparing online modalities to each other or to hybrid classrooms. As one of the first influential meta-analyses in this category, Shachar and Neumann (2003) meta-analyzed 86 studies between 1990 and 2002 that directly compared a DE course to an in-person course. In 66% of their studies, DE outperformed in-person instruction models (average effect size of *d* = 0.37; Shachar & Neumann, 2003); however, criteria for inclusion as “online” were not articulated and could bias their findings.

The following year, Bernard et al. (2004) performed a similar meta-analysis comparing DE with “classroom instruction,” except that they expanded the search window to include studies dating back to 1985. In addition, the authors set criteria where their DE classes for study inclusion had to report less than 50% in-person instruction and potential studies had to measure one of the following outcomes: achievement, attitudes, or retention. While these criteria increased transparency beyond the Shachar and Neumann (2003) meta-analysis, these criteria still lumped hybrid and fully online courses as equivalent DE delivery methods. They also made a further distinction of whether the DE courses included only asynchronous interactions or at least some synchronous interactions. For achievement, Bernard et al. (2004) found an overall slightly positive effect of DE over in-person instruction; however, the effect sizes and directionality differed by mode with the majority of effect sizes for synchronous DE being negative, while asynchronous effects were positive. For attitudes, Bernard et al. (2004) found that students in DE courses had significantly poorer attitudes, especially in a synchronous format. And lastly, retention had a slight but significant negative effect favoring in-person instruction but with wide variations in effect sizes between studies (Bernard et al., 2004).

Similarly, Zhao et al. (2005) analyzed 51 studies published between 1966 to 2002 including a wide range of DE methodologies all classified as education at a distance, yet they controlled for several moderators in their analysis to quantify their influence on effect sizes including the year of publication, whether the researcher was also the instructor, the instructor’s involvement in the course, level of student (high school or college), and whether or not any instruction occurred face-to-face. Overall, two-thirds of their included studies demonstrated a positive effect favoring DE over in-person instruction (Zhao et al., 2005). However, when compared with the magnitude of the one-third that had a negative effect, the overall effect size was zero. Zhao et al. (2005) describe that higher effect sizes were associated with analysis factors (i.e., articles published post-1998), factors of the study participant populations (i.e., high school students were higher than college students), curricular design factors (i.e., greater involvement of the instructor in the DE course, greater proportion of in-person instruction), and potential bias factors (i.e., if the authors was the instructor of the DE course). Again, this analysis is complicated by the wide range of online methodologies included in the analysis.

In 2009, the US Department of Education conducted a thorough review of online learning (Means et al., 2010), and although they included many forms of DE, their analysis tightly controlled for fully online courses versus blended or hybrid courses. They analyzed 99 studies conducted between 1996 and 2008 that compared in-person instruction to some form of DE. Of the 45 with sufficient data, in-person instruction was compared to fully online instruction in 27 cases and to blended formats in 23 cases (Means et al., 2010). They concluded that overall, online conditions performed modestly better than in-person, blended courses significantly outperformed in-person courses (g =  + 0.35), yet fully online courses had no significant advantage over in-person courses. However, the authors point to the varied conditions of the curricula (time-on-task, pedagogical approaches, etc.) for this modest improvement by delivery method; furthermore, this analysis showed that the more similar the curricula were in the comparison, the smaller the effect size (Means et al., 2010).

From these meta-analyses, we can see a positive trend for online instruction, with wide variety in effect sizes. However, little, if any, information is known about the comparison between DE and flipped classrooms. This suggests the need for more controlled and rigorous approaches to evaluating DE (Lee, 2017). In this study, we compare two courses that are practically equivalent in terms of design, content, implementation, narrative, assessment, and even instructor affect, utilizing a blended, technology-enhanced flipped classroom as our in-person and a fully online (asynchronous) comparison course.

### Problem Statement

Online education is increasingly being incorporated into the undergraduate experience, and the current state of education demands that we offer this delivery method, especially in light of recent events (i.e., a global pandemic that has forced the closure of all institutes of higher education during Spring 2020). However, research has failed to adequately test whether curriculum-equivalent courses in an in-person and asynchronous online modality produce disparate learning outcomes. In a tightly controlled study of nearly equivalent courses, we aimed to investigate whether a baseline model of an asynchronous online course (where equivalent curricula are moved directly online, and instructor/peer interaction was limited to asynchronous communication that required student initiation) leads to equivalent student performance.

## Materials and Methods

### Ethics Statement

Permission to do research with human subjects was acquired through the Institutional Review Boards at the participating institutions. All participants completed a formal written consent prior to collecting data.

### Subjects

Subjects were recruited from two institutions in the Western United States with varying academic diversity prior to the COVID pandemic as part of a push for asynchronous online offerings by the private institution and as an ongoing effort for these offerings at the public institution. The first institution is a large private university (~ 30,000 students) with an incoming freshman average high school grade point average (GPA) of 3.85 and an average American College Test (ACT) score of 29.2. The other is a large public university (~ 32,000 students) with open enrollment whose incoming freshman average high school GPA was 3.27 and had an average ACT score of 23. Students were recruited from introductory biology courses that fulfill a general education requirement for non-biology majors.

The two modalities of courses (asynchronous online and in-person) were taught by two different instructors, one at the private university and one at the public institution mentioned previously. Between these two institutions, 209 undergraduate students participated in the study. At the private institution, there were 115 enrolled in the in-person treatment and 37 enrolled in the online treatment. At the public institution, there were 29 enrolled in the in-person treatment and 28 enrolled in the online treatment.

### Measures of Group Equivalence

To control for potential group non-equivalence, we assessed the students’ general scientific reasoning skills using the *Lawson Classroom Test of Scientific Reasoning* (LCTSR; Lawson, 1978, ver 2000). We chose this instrument as a measure of group equivalence and to use as a potential covariate if our data showed the groups to be non-equivalent because scientific reasoning has been shown to be highly correlated with performance in science classes (e.g., Johnson & Lawson, 1998). The LCTSR is a content-independent test of basic reasoning skills, including proportional, combinatorial, probabilistic, correlational, and hypothetico-deductive reasoning, as well as the ability to identify and control variables; validity and reliability have been well established (Lawson et al., 2000). The LCTSR was delivered at the beginning of the semester, prior to learning any course content.

### Course Structures

We designed two courses, an asynchronous online version and an in-person version, to have identical objectives, assessments, and learning experiences with the only variation being that one course offered all elements asynchronously online while the other was offered partially in-person. Thus, we are testing both the effects of online delivery as well as the effects of asynchronicity where instructor interaction was via email and/or in-person office hours and peer interaction during the learning process was lacking, i.e., our comparison group is as fully online, fully asynchronous course, as is traditionally offered in many online instructional environments. Given that technology is a delivery tool for instruction, rather than a pedagogical technique itself (Jensen et al., 2012), we feel that the main effect we are testing is the lack of peer/instructor interaction during instruction that is implicit in an asynchronous online course.

The format we chose for the in-person course was a flipped classroom in which students attained content entirely asynchronously at home prior to coming to class and then practiced applying that content during in-class activities (O'Flaherty & Phillips, 2015). As a technology rich, blended format, a flipped classroom seemed to be the best comparison to an entirely online course since half of the course content would be identical and delivered using the same asynchronous online modality. The flipped classroom is a well-researched effective method of teaching an active, student-centered classroom (e.g., Jensen et al., 2015; Strayer, 2012; Tucker, 2012; Tune et al., 2013). We expected that using a flipped classroom, as opposed to a traditional lecture classroom, as our in-person comparison, increased the content equivalence for the courses. We designed the curriculum using a constructivist approach patterned after the 5-E learning cycle (Engage, Explore, Explain, Elaborate, and Evaluate; Bybee, 1993). We divided instruction into two components: content attainment, where students learn the initial content and build conceptual understanding through prepared videos and formative assessments; and concept application, where students practiced applying the content to new contexts through interactive exercises to further solidify and broaden their conceptual understanding and demonstrated this knowledge in summative assessments. Content attainment was completed asynchronously online for both treatment groups. In the in-person flipped treatment, the concept application occurred synchronously in-person; in the online treatment, this occurred asynchronously online, as described below.

#### Content Attainment

Video lectures are a typical method used to deliver content online, especially in a “flipped” classroom (Abeysekera & Dawson, 2014) and were found to be superior to textbook-style readings covering the same content (Jensen et al., 2018). Scripted, screencast video lectures were created by two professors at one of the institutions to engage students and allow them to explore and explain the content for each unit. Every section used the same set of video lectures, regardless of whether it was an asynchronous online or in-person flipped format. The video lectures were constructivist, in that they presented students with puzzling phenomena, identified common misconceptions, and explained the construction of concepts using data, demonstrations, and scenarios. Students did not, however, interact directly with the media. Each of the 41 days of the semester, on a usual Monday-Wednesday-Friday schedule, consisted of two to six short videos for students to watch, each ranging from 5 to 12 min in length. These videos contained all the content disseminated to students and were not supplemented with additional texts or readings. Students had access to these videos, via an unlisted YouTube link, at all points during the semester and could review them as desired. A simple, one-question yes/no response quiz was included at the end of the videos to verify student participation on an honor system.

Following each “day” of content attainment, students were required to complete a formative assessment (i.e., a quiz) over the material. Students were instructed to take the quiz on their own without assistance from notes on their first attempt, in order to self-evaluate their learning from watching the video(s) for that day. Students were shown their score but not which questions were marked incorrect. After their first attempt, they were encouraged to retake the quiz, using notes and other resources, an unlimited number of times until they achieved their desired score. Students’ highest obtained score was recorded for grading purposes. There was no penalty for the number of attempts, further encouraging students to use the initial attempt as a formative assessment and then to continue attempts until they achieved their personal goal. Initial quiz scores and total number of attempts were recorded for analysis purposes.

#### Concept Application

In line with the 5-E learning cycle, following content attainment, both in-person and online courses completed the same elaboration activities. These activities were designed as problem sets that asked students to apply the knowledge they just gained to novel situations. In the in-person sections, students completed these activities during class time, working together in groups with instructor facilitation. In the fully online section, students completed these activities online without peer or instructor facilitation using the survey software Qualtrics (XM) and the universities’ learning management systems (i.e., Canvas). The content and tasks required of students in these activities were identical between treatments. Online students had access to instructors and teaching assistants to seek assistance with these activities via email or in-person during office hours. However, online students had to solicit this help on their own, while the in-person students interacted with peers and their instructor three times a week in a face-to-face environment.

#### Summative Assessments

At the completion of each unit, which were approximately 2 weeks in length, there was a summative Unit Exam (8 in total) to evaluate students’ deep conceptual understanding and ability to apply the learned concepts. Exams were identical for all treatments. Exams were open-note and were administered online. Each had an average of 14.6 multiple-choice items per exam, and an hour time limit encouraged students to adequately prepare and discouraged them from looking up answers in real-time. These exams were written by the instructors at higher levels of Bloom’s taxonomy (Bloom, 1984) such that answers were not easily accessed online, but critical thinking and deep conceptual understanding was required. At the conclusion of the course, each student took a closed-book final exam in which they had 2 h to answer 35 multiple-choice, high-level Bloom’s questions. In the in-person treatment, the final was administered in-person at the public institution or in the university testing center at the private institution. In the online treatment, the final was administered through the university testing center at the public institution and through a proctoring system online where students were required to use a webcam and the program locked down student computers except for access to the exams at the private institution. All sections, regardless of the treatment, were allowed an optional 8.5″ × 11″ sheet of notes on the final exam.

### Dependent Measures

Each treatment was evaluated by comparing scores of the content attainment quizzes, unit exams, and the final exam. For each student, the first attempt of all 41 content attainment quiz scores were averaged to represent a measure of each students’ initial understanding and attainment of knowledge from the same videos, which all treatments were required to watch online. Additionally, we averaged the number of attempts each student took on these content attainment quizzes across the entire semester. Similar to the quizzes, all individual students’ exam scores were averaged to yield an average exam score for each student. The final exam was reported as a total score. These exams were used to test content mastery and retention. Scores were compared using ANCOVA with LCTSR scores as a covariate, with in-person or online as the between-subjects factor and scores on each assessment as within-subjects factors.

### Academic Background Factors

The online courses at both institutions were intended for and offered to full-time, daytime students. They were not intended to be part of an independent study curriculum or to be catered to non-traditional students. However, students were self-selected into each modality. Unfortunately, we did not have permission or means to collect disaggregated academic background data for students participating at the public institution. As a small case study, with a portion of our data, we were granted permission to gather academic background factors from students at the private institution, including academic standing when taking the course (good or poor; poor being defined as a GPA that drops below 2.0 in a given semester), academic status (freshman, sophomore, junior, or senior), and major (STEM or non-STEM). These factors were used in a linear regression analysis to determine their effects on performance.

## Results

### Scientific Reasoning Ability

LCTSR scores did not meet the assumptions for parametric tests; thus, non-parametric alternatives were used. To determine group equivalence, we compared LCTSR scores between treatment groups. At each institution, in-person and online students had statistically equivalent LCTSR scores indicating that groups were equivalent according to this measure (M_Private in-person_ = 78.6, M_Private Online_ = 68.5, *U* = 1595.00, *z* = 1.50, *p* = 0.13; M_Public in-person_ = 58.9, M_Public Online_ = 53.4; *U* = 187.00, *z* = 1.07, *p* = 0.28). In addition, students at the private institution overall had higher scores than students at the public institution [*U* = 5164.00, *p* < 0.001].

### Content Attainment Quizzes

**Fig. 1:**
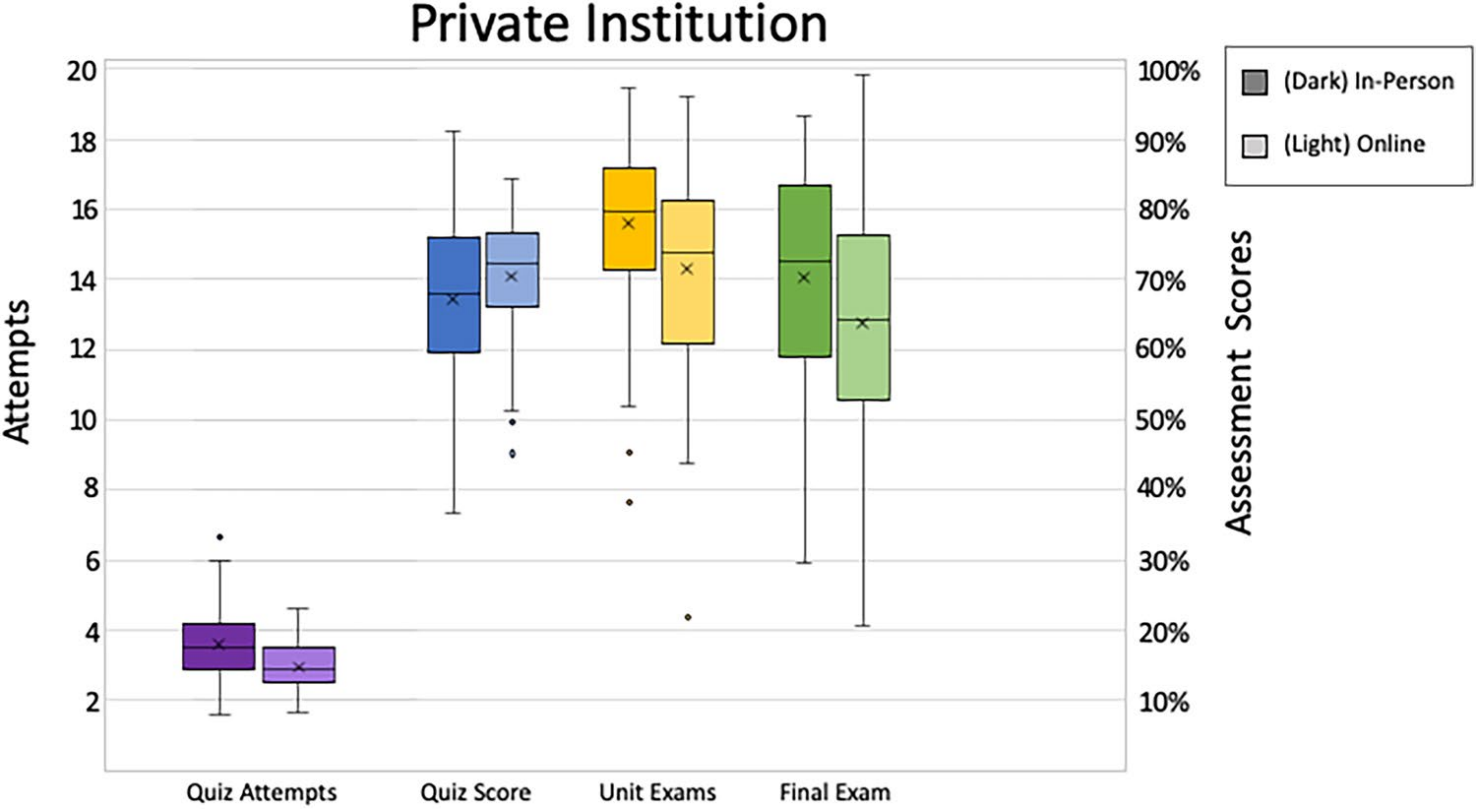
In-person and online students’ first attempt scores in private institutions

**Fig. 2:**
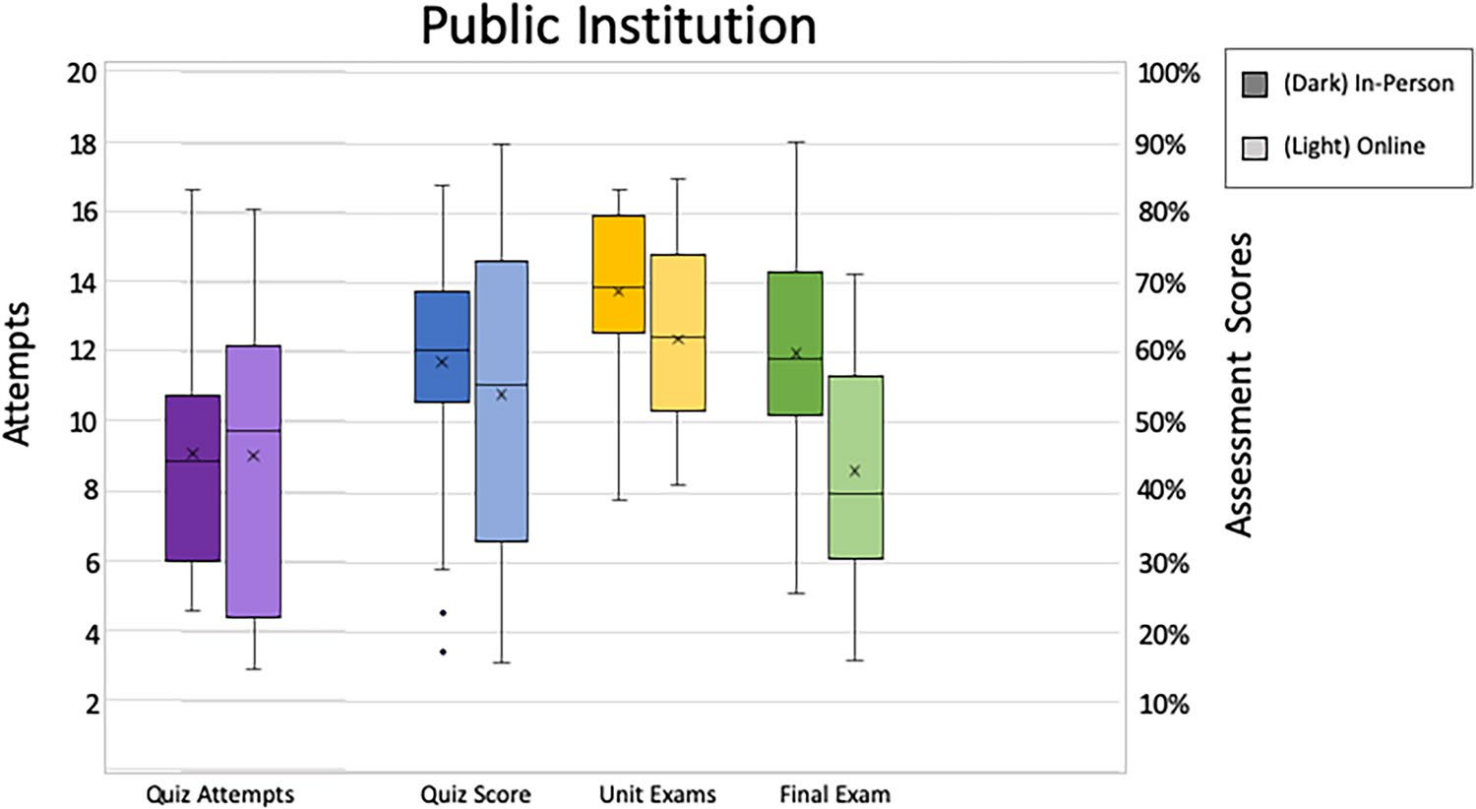
In-person and online students’ first attempt scores in public institutions

We used students’ first attempt of the content attainment quiz score to indicate initial “content learning” from the video lecture activities. Again, non-parametric tests were used due to violation of assumptions. At the private institution, in-person and online students’ first attempt scores were statistically equivalent. [M_Private in-person_ = 67.2, M_Private Online_ = 70.4, *U* = 2492.00, *z* = 1.57, *p* = 0.118; Fig. 1]. The same pattern was seen at the public institution, [M_Public in-person_ = 58.6, M_Public Online_ = 54.0, *U* = 353.00, *z* = 0.85, *p* = 0.40; Fig. 2]. Overall, content attainment quiz scores were higher at the private institution than at the public institution [M_Private Total_ = 68.0, M_Public Total_ = 57.9; *U* = 5956.00, *z* = 4.17*, p* < 0.001].

### Content Attainment Quiz Attempts

As a further assessment of content attainment, we looked at the number of attempts students took on these content attainment quizzes, again using non-parametric approaches. More attempts could indicate less initial learning or more investment in the learning process. We found at the private institution, online students made fewer attempts than in-person students [M_Private in-person_ = 3.6, M_Private Online_ = 2.9, *U* = 1250.50, *z* = 3.77, *p* < 0.001; Fig. 1]. There were no statistical differences in the number of attempts at the public institution [M_Public in-person_ = 9.1, M_Public Online_ = 9.0; *U* = 409.00, *z* = 0.05, *p* = 0.96; Fig. 2]. We also found a significant difference in the number of attempts between institutions. Overall, students at the public institution made notably more attempts on content attainment quizzes than the students at the private institution [M_Private Total_ = 3.4, M_Public Total_ = 9.0; *U* = 536.00, *z* = 9.75, *p* < 0.001].

### Unit Exams

Unit exams were the summative assessment for each unit in the course. Scores did not meet assumptions for parametric tests, so nonparametric alternatives were used. At the private institution, students in the in-person treatments outperformed students in the online treatment [M_Private in-person_ = 78.1, M_Private Online_ = 71.5, *U* = 1619.00, *z* = 2.18, *p* = 0.03; Fig. 1]. Similarly, at the public institution, in-person students significantly outperformed online students [M_Public in-person_ = 68.8, M_Public Online_ = 62.0, *U* = 269.00, *z* = 2.19, *p* = 0.03; Fig. 2]. Overall, students at both institutions, when instruction modality was pooled (in-person and online scores combined), had statistically equivalent scores [M_Private_ = 76.5, M_Public_ = 66.4; *U* = 6408.00, *z* = 5.33, *p* < 0.001].

### Final Assessment

The final assessment was a comprehensive exam and served as the students’ summative assessment for the entire course. Again, nonparametric alternatives were used. At the private institution, students in the in-person treatment significantly outperformed students in the online treatment [M_Private in-person_ = 70.2, M_Private Online_ = 63.8, *U* = 1573.00, *z* = 2.38, *p* = 0.02; Fig. 1]. At the public institution, the same pattern emerged, [M_Public in-person_ = 60.0, M_Public Online_ = 43.2, *U* = 187.5, *z* = 3.49, *p* < 0.001; Fig. 2]. Overall, students at the public institution performed lower on the final assessment than the students at the private institution, when instruction modality was pooled [M_Private_ = 68.7, M_Public_ = 53.6; *U* = 6543.50, *z* = 5.68, *p* < 0.001].

### Predictive Factors

**Fig. 3:**
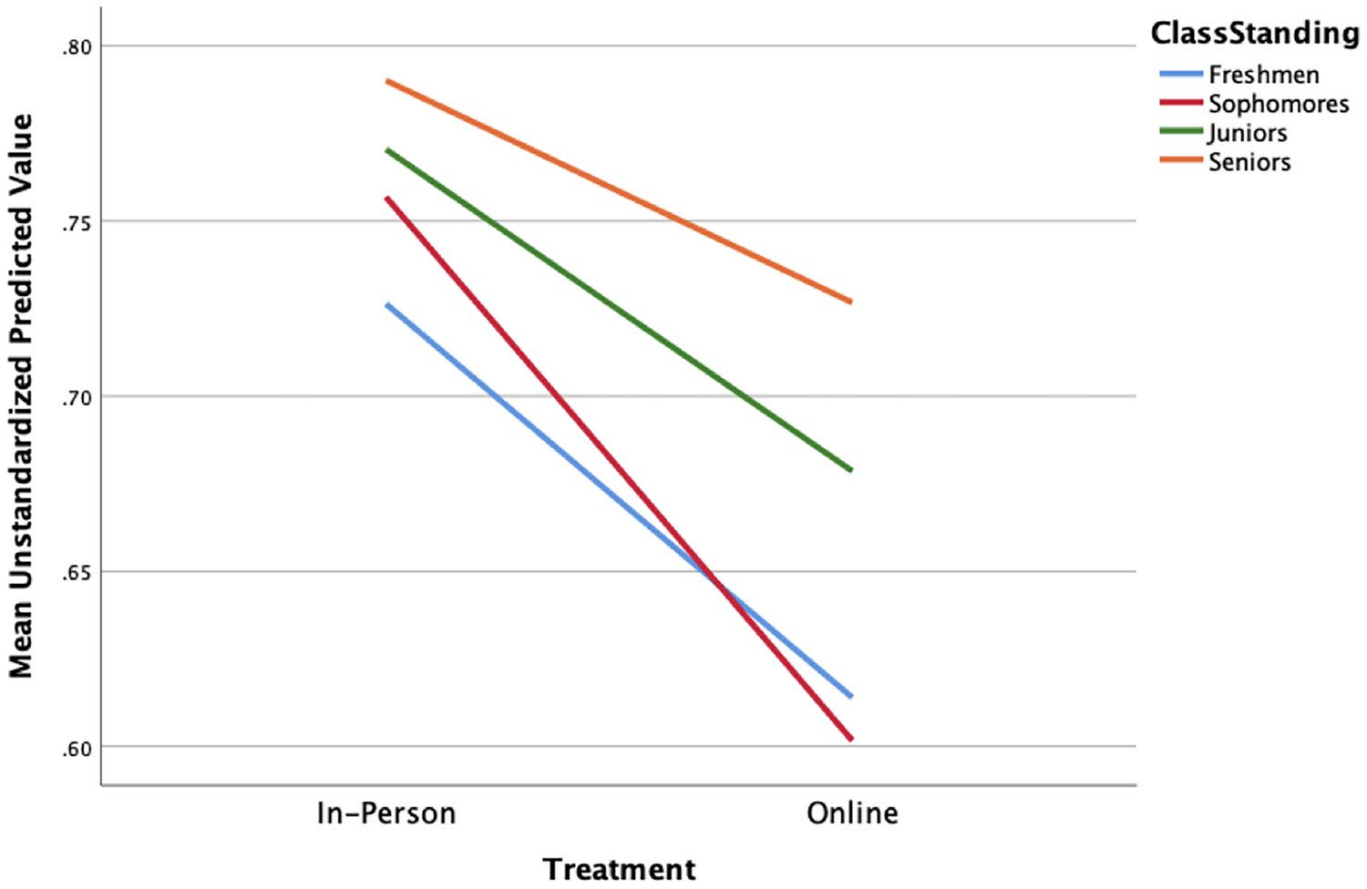
Significant interaction between treatment and class standing with the online treatment

We ran a multiple linear regression on the private institution data subset on summative assessments (unit and final exams) to determine whether the inclusion of academic background factors could explain the gap in performance between in-person and online instruction. These data met all assumptions of linear regression (i.e., independence of observations, linearity, no homoscedasticity, no multicollinearity, no outliers, and normality). For average unit exam scores, the model with treatment, academic standing, academic status, major, academic standing × treatment, academic status × treatment, and academic status × major accounted for 24.9% of the variability in performance, a small effect, F(7,634) = 29.72, *p* < 0.001. There was a significant interaction between treatment and class standing with the online treatment appearing to disproportionately decrease scores among sophomores (see Table 1 and Fig. 3).

**Table 1:** Summary of multiple regression analysis for unit exam scores

Variable	*B*	*SE*_*B*_	*b*	*t*	*p value*
Intercept	.550	.022			
Academic standing	.160	.020	.341*	8.13	< .001
Class standing	.018	.006	.135*	3.19	.001
Major	.045	.010	.164*	4.27	< .001
Treatment	-.212	.051	-.601*	-4.16	< .001
Academic standing × treatment	.028	.036	.075	.801	.424
Class standing × treatment	.032	.014	.288*	2.34	.020
Major × treatment	.029	.025	.054	1.16	.249

* *p* < .05, *B,* unstandardized regression coefficient; *SE*_*B*_, standard error of the coefficient; *b*, standardized coefficient

**Fig. 4:**
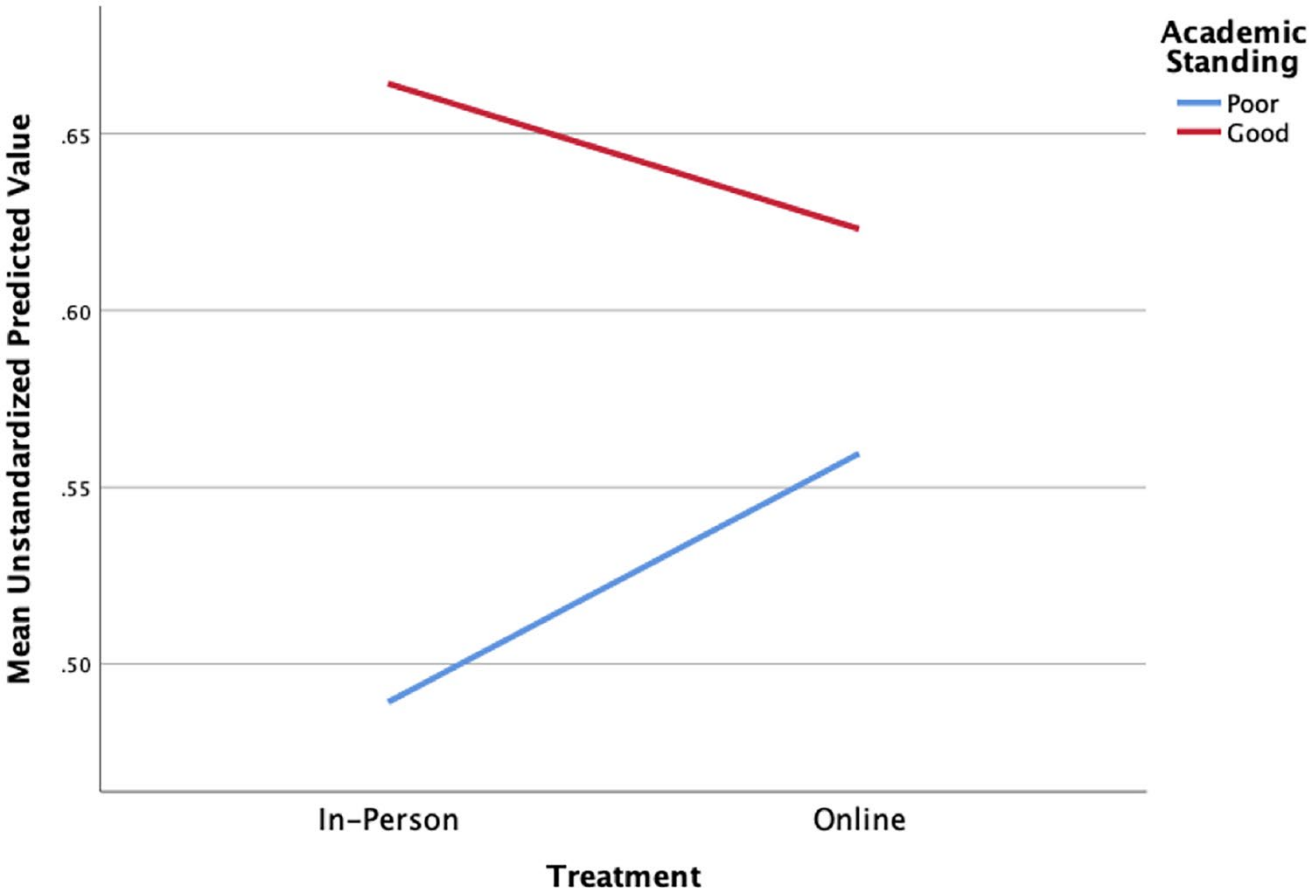
Significant interaction between treatment and academic standing with those in poor/good academic standing

For final exam performance, the model accounted for 15.2% of the variability in performance, a very small effect, F(7,634) = 16.09, *p* < 0.001. A significant interaction was seen between treatment and academic standing with those in poor academic standing scoring higher in an online condition and those in good academic standing scoring higher in in-person classes (see Table 2 and Fig. 4).

**Table 2:** Summary of multiple regression analysis for final exam scores

Variable	*B*	*SE*_*B*_	*b*	*t*	*p value*
Intercept	.402	.028			
Academic standing	.176	.025	.311*	6.96	< .001
Class standing	.034	.007	.210*	4.67	< .001
Major	.053	.013	.159*	3.91	< .001
Treatment	.019	.066	.044	.29	.776
Academic standing × treatment	-.113	.046	-.246*	-2.48	.013
Class standing × treatment	.002	.017	.019	.141	.888
Major × treatment	.021	.033	.032	.643	.521

* *p* < .05, *B*, unstandardized regression coefficient; *SE*_*B*_, standard error of the coefficient; *b*, standardized coefficient

## Discussion

In this study, our intention was to compare student outcomes between fully asynchronous online and partially asynchronous online (flipped) introductory biology classes using two differentially achieving student populations. Our novel contribution, amid the prolific literature comparing online to in-person instruction, was that we maintained identical content, learning objectives, activities, and assessments in both treatments. In addition, we analyzed various academic background factors to determine their effect on performance between modalities.

### Formative Assessments

In our study, both treatments watched the same videos and took the same formative assessments asynchronously. Therefore, it was not surprising that we found no differences in initial content attainment quiz scores between the in-person and asynchronous online modalities at either institution. Interestingly, students in the in-person section at the private institution used more attempts to achieve a final score they desired than in the online treatment. These behavioral differences by our participants mirror self-regulation strategies also observed by Barak et al. (2016), where in-person learners exhibit greater self-discipline to achieve their goals. Alternatively, online students may have been more efficient with their time, identified as a critical self-regulated learning strategy in online education (Broadbent & Poon, 2015), and judiciously use less attempts to achieve their goals. At the public institution, however, students made far more attempts in both treatments than at the private institution, further hinting at this population’s lower level of preparedness also indicated by the lower Pre-LCTSR scores. In general, the lack of difference in performance on formative assessments, for each population, across treatments indicates the equivalence of learning content gained by the videos. Therefore, any subsequent difference arose because of variation in modality of delivery of the learning experience when applying the content (i.e., in an online, asynchronous, individualized attempt or in an in-person, synchronous, collaborative attempt).

### Summative Course Learning

We observed that in-person sections scored higher on summative assessments than asynchronous online sections at both the private (8% higher on unit exams, 3.7% higher on final exam) and public (6% higher on unit exams, 11.4% higher on final exam) institutions. In addition, we saw that the private institution outscored the public institution on both summative assessments in both modalities, further confirming the difference in academic preparation between a highly selective and open-enrollment institution (Jensen et al., 2018). It also supports several recent studies that suggest that some modalities of online education lead to lower learning gains than face-to-face instruction (e.g., Gundlach et al., 2015; Johnson & Palmer, 2015; Poelmans et al., 2018).

Our online course was created with the strategy to serve as a direct comparison to the flipped classroom method by controlling for other factors that have complicated studies in the past. It is possible that other additional materials, tools, or support that are not present in our online design might allow for improved performance, one major component being synchronous peer or instructor support, but that is not the purpose of this study. Rather, one can think of our online designed course as a baseline model to allow us to maximize the comparison to the flipped course, allowing us to pinpoint the specific differences in performance in an asynchronous fully online course without complications of extracurricular materials or support that is often typical and recommended in online courses. In our baseline model (i.e., asynchronous), online students consistently perform lower than in-person flipped classroom students suggesting that online environments where no synchronous interaction occurs are less effective at fostering learning. However, to the extent possible, comparisons that equalize the content, materials, activities, support, etc. are the most appropriate kinds of studies to evaluate modality of delivery on student success.

We wish to offer some interpretation of causal mechanisms for our findings. In other words, what is happening during the in-person portion of the flipped modality that might be making the difference? We have hypothesized several reasons for this difference as there are some aspects of in-person classes that cannot be easily replicated in an asynchronous online course. These include the addition of faculty-student interactions, peer-peer interaction, on-demand instructor scaffolding, and personal accountability encouraged by face-to-face interactions.

Faculty interactions, as well as peer interactions, help students to ask questions, get clarification, and receive immediate feedback during the learning process (in the case of our in-person class, it was during the concept application phase) (Joosten et al., 2019; Martin et al., 2019). The Social Presence Theory suggests that the learning process is more than just the pedagogical interventions and content presented, but is also heavily influenced by the nature of interactions and social context in which the learning takes place (Bandura, 1985; Lee, 2014; Li et al., 2016). This theory would suggest that students who have relationships with and interact directly with their professors would perform better than students that do not have these interactions. In addition, peer-peer interactions, which are not easily implemented in an asynchronous online course as students may never even meet each other, may also facilitate learning in an in-person environment. With that lacking in an online course (and not specifically built in to our online modality), it would stand to reason that it may affect the students’ overall performance.

On-demand instructor scaffolding is when the teacher adds support for students that leads to the enhancement of learning and assists students in achieving mastery (Wood et al., 1976). This takes place very intentionally when the teacher builds on the students’ personal experiences and prior knowledge as they are attaining the new content and skills. This is possible when the instructor is teaching content in real-time and more difficult when lessons are in an asynchronous online modality. Even though some individual scaffolding was designed in the online apply activities, it is likely not as constructive as in-person instructor scaffolding. Thus, a lack of on-demand instructor scaffolding could likely explain differences in performance.

Finally, the level of accountability that naturally comes when students are required to attend a specific class time and participate in the class discussions does not exist when courses are asynchronous and online. Although our course built in regular due dates for a small level of accountability, and students were often reminded of these due dates, students could choose to complete course requirements whenever and wherever they chose within the time frame. This can easily lead to procrastination, less attention, and a smaller level of accountability felt on the side of the student. Previous research has shown that students in online courses do exhibit lower accountability and that the success rates (e.g., students who complete and/or pass the course) of online courses are lower than in-person courses (Hedges, 2017; Sapp & Simon, 2005; Waschull, 2001).

The other side to this coin is that in-person classes should take advantage of online tools. It is possible that one of the reasons that many of the previous studies supported no difference or better performance of online classes is that they were being compared to in-person classes that did not take advantage of online resources and tools that enhance the learning process. The flipped or blended modality of instruction tends to provide a good mix of in-person and online experiences and activities. Thus, this highlights another reason that this comparison is a more realistic approach to evaluate the efficacy of two well-designed courses, asynchronous online and hybrid in-person.

### Public vs. Private Institution Performance

Overall, we found that the private institution performed better than the public institution across both modalities, but only on summative assessments. However, the difference between online and in-person modalities at each institution showed a similar trend. Testing this treatment in both student populations gives us insight and allows our research to have more broad implications for schools across the country. Overall, schools with less stringent admissions requirements may consider offering more scaffolding to their online courses to help less-prepared students (Gregory & Lampley, 2016). One should certainly keep in mind that online courses can provide greater access to higher education for those students who must balance family, work, and school responsibilities (Aslanian & Clinefelter, 2013; Brinkerhoff & Koroghlanian, 2007; Jaggars, 2014).

### Variations in Academic Background

We found that academic background did not help explain the difference in the performance gap between in-person and asynchronous online treatments in the private institution subset. Potentially other background factors, that we did not measure, may help explain differences in performance between students in in-person and online courses. For example, we were unable to include potentially confounding variables that may explain the performance gap between modalities, like personal circumstances, goals, previous experience in online learning, and motivation. One study, for example, found that the top three reasons students chose to take an online course were first, the assumption that online learning is easier; second, they “lacked time to attend regular classes”; and third, online courses enabled them to “combine college with family responsibilities” (Brown, 2012, p. 41). It is possible that students who have time-consuming commitments or who are seeking a less difficult educational experience put less effort into coursework than traditional students who are singularly focused on college and that the former are more likely to enroll in online courses than the latter. Indeed, “[s]elf-selection is one of the major issues while comparing the performance of the students in online and face-to-face classes” (Pathak, 2019, p. 16). Future research should account for selection bias to determine what, if any, role it has in the disparity between educational outcomes.

## Conclusion

Several lessons can be learned from this work. Perhaps as a whole, both public and private institutions should be more thoughtful as more pressure is applied to move to increased online course offerings. This study highlights the importance of performing content equivalent comparisons in order to objectively measure student success in online and in-person environments. In addition, it appears that asynchronous online courses require additional scaffolding and instructional features that are implicit in an in-person class in order to improve performance, such as synchronous peer and instructor scaffolding. Future studies should investigate ways to simulate the missing scaffolding (e.g., weekly recitations or built-in tutorials of frequently asked questions or common misconceptions) and peer interaction. Perhaps to address the lack of peer-to-peer collaboration in online classes, asynchronous or synchronous chats between peers could be used (see Singhal, 2020, for an example). Also, to address a form of advanced scaffolding or on-demand instructor help, it may be possible to embed tutorials into actual homework assignments that directly address common misconceptions at the moment they might occur. With the addition of varying forms of scaffolding, we may be able to improve online courses from the baseline version tested in this study. This study stands as a strong example where asynchronous online education is inferior to the university model of students going to classes to be with their instructors and should serve as a starting point for more content-controlled comparison to get at the heart of best practices in online education.

## Data Availability

Data will be available on BYU ScholarsArchive (scholarsarchive.byu.edu) upon publication.
